# Salivary Cortisol Levels in Patients with Oral Lichen Planus—A Pilot Case-Control Study

**DOI:** 10.3390/dj7020059

**Published:** 2019-06-01

**Authors:** Ivana Skrinjar, Valentina Vidranski, Bozana Loncar Brzak, Danica Vidovic Juras, Ana Andabak Rogulj, Vlaho Brailo, Vanja Vucicevic Boras

**Affiliations:** 1 Department of Oral Medicine, University Clinical Hospital Center Zagreb, 10000 Zagreb, Croatia; skrinjar.ivana@gmail.com (I.S.); dvjuras@gmail.com (D.V.J.); brailo@sfzg.hr (V.B.); borasvanja@yahoo.com (V.V.B.); 2 Department of Nuclear Medicine and Oncology, University Clinical Hospital Sisters of Mercy, 10000 Zagreb, Croatia; vvidranski@gmail.com; 3 Department of Oral Medicine, School of Dental Medicine University of Zagreb, 10000 Zagreb, Croatia; anaandabak@gmail.com

**Keywords:** oral lichen planus, saliva, cortisol, stress

## Abstract

It is known that cortisol level increases in stress situations. The aim of the study was to measure the levels of salivary cortisol in patients with oral lichen planus (OLP) and healthy controls. This was a case-control pilot study which included seven patients with reticular (non-symptomatic) OLP, eight patients with atrophic/erosive (symptomatic) OLP, and nine healthy controls. We hypothesized that patients with an atrophic/erosive type of OLP have higher levels of cortisol compared to patients with the reticular type of OLP and healthy controls. In each participant, unstimulated saliva was collected in order to determine cortisol levels by using commercially available ELISA kit. Our results have shown no differences between levels of salivary cortisol in OLP patients and healthy controls. We can conclude that further research with a larger number of OLP patients is needed to determine the correlation between OLP and stress.

## 1. Introduction

Oral lichen planus (OLP) is an autoimmune disease with a chronic feature, and a still unclarified etiopathogenetic and is predominant in middle-aged and elderly women [1]. Except for the oral mucosa, lichen planus can appear on other mucous membranes and skin. A feature of skin lesions of lichen planus is itching and self-limitation contrary to oral lesions which are usually chronic and can lead to morbidity [2]. The diagnosis of OLP is mostly achieved by clinical examination and confirmed by histopathological analysis.

An association between OLP and psychological disorders have been well documented [3]. It is considered that psychological factors are involved in the pathogenesis of OLP. Higher levels of anxiety and depression with increased sensitivity to psychological disorders were found in comparison to healthy controls which resulted in the need for oral lichen planus to be investigated as a psychosomatic disease [4]. Despite the higher levels of psychological stress and anxiety presented by OLP patients, it is still unclear whether the psychological factors are involved in the etiology of OLP or they are a result of the morbidity associated with the disease [5].

Reactions to stress are associated with elevated secretion of various hormones including glucocorticoids which enhance mobilization of energy sources and adapt the individual to new circumstances [6].

Cortisol concentrations rise in response to physical or emotional stress and are disrupted by shift work and sleep deprivation. Salivary measurements have the advantage of being noninvasive, whereas blood sampling may be stressful and artificially elevate cortisol levels [7]. Advances in laboratory techniques have enabled the measurement of salivary cortisol and cortisone to a high level of sensitivity and specificity.

The aim of the present study was to measure differences in salivary cortisol levels in patients with reticular (non-symptomatic) and atrophic/erosive (symptomatic) forms of OLP and healthy controls in order to reveal the correlation between OLP and stress. We hypothesized that patients with an atrophic/erosive type of OLP have higher levels of cortisol compared to patients with a reticular type of OLP and healthy controls.

## 2. Materials and Methods

This study was approved by the ethical committee of the University Hospital Center Zagreb (N0 02/21, 11/22/2018) and each participant signed informed consent according to the Declaration of Helsinki.

This was a case-control pilot study which included 15 patients with OLP, seven patients with the reticular type of OLP and eight patients with the atrophic/erosive type. The mean age of the patients in the study groups was—reticular OLP—53 (range 40–61); atrophic/erosive OLP—56 (range 40–58), and the control group—55 (range 38–60) years. Three patients were male (one in each group) and 12 were women. Inclusion criteria were patients with newly diagnosed OLP which was clinically diagnosed by an oral medicine specialist and confirmed by histopathological examination. Patients did not have skin or any other lesions or any other oral disease. Excluding criteria were patients taking anxiolytics, anticonvulsants, antidepressants, or hormonal therapy which could affect the cortisol levels.

The control group included nine healthy individuals without any systemic or oral disease and not taking any medications which were age and sex matched with the study groups.

In order to determine the cortisol levels, unstimulated saliva was collected from each participant between 9 AM and 10 AM in order to minimize diurnal variability by spitting into grading tubes. The patients were instructed not to brush their teeth or ingest or place any substance in their mouth for one hour before saliva collection. All samples were immediately frozen. Saliva samples were defrosted completely at room temperature (20–23 °C) on the day of the assay, vortexed, and centrifuged for 10 min at 1500× *g* at 4 °C on a Hettich ROTINA35 centrifuge (Hettich, Germany). Analysis of saliva was performed using a competitive ELISA (enzyme-linked immunosorbent assay) kit for the quantitative measurement of cortisol (µg/dL), (Salimetrics, LCC, State College, PA, USA). The test had an analytical sensitivity of 0.007 ug/dL and an assay range of 0.012–3.000 ug/dL. The plate was read at 450 nm on a plate reader VIKTOR2 Wallac 1420 Multilabel Counter (Perkin Elmer, Massachusetts, USA). The amount of cortisol enzyme conjugate detected was inversely proportional to the amount of cortisol present in the sample.

Statistical analysis was performed by the MedCalc statistical software version 18.10.2 (MedCalc Software, Belgium). As the tested variables did not have a normal distribution, the differences between salivary cortisol levels were calculated by the non-parametric Kruskal–Wallis test, with a significance level 0.05 (*p* < 0.05).

## 3. Results

Regarding sex and age, there were no significant differences between groups. Although salivary cortisol levels were highest in patients with reticular OLP, our results have shown that there were no significant differences between salivary cortisol levels in patients with the atrophic/erosive or reticular type of OLP as well as healthy controls (*p* > 0.05) (Table 1).

## 4. Discussion

Results from the previous studies regarding salivary cortisol levels in patients with oral lichen planus are controversial. According to recent systematic review [8], five studies have shown elevated salivary cortisol levels in patients with OLP compared with healthy controls [9,10,11,12,13]. Results of other studies have shown no differences between salivary cortisol levels in patients with OLP and healthy controls or elevated salivary cortisol levels in healthy controls [14,15,16,17]. Rödström et al. [14] concluded that there was no support for a reduced ability of OLP patients to suppress an immune through the induction of cortisol in connection with experimental stress. In their study patients with an erosive type of OLP were included. Girardi et al. [15] stated that cortisol and dehydroepiandrosterone (DHEA) levels did not differ between groups, which does not support any neuroendocrine etiology for OLP. They included all patients with OLP in one group, with no distinction between reticular (non-symptomatic) and atrophic/erosive (symptomatic) types of OLP. These results of no differences between OLP patients and healthy controls are in concordance to ours. Opposite to these results, Shah et al. [11] found a positive correlation between depression, anxiety, and stress with salivary cortisol levels in patients with OLP.

Different results may be explained by a different methodology used in different studies. Selection of the patients and also different methods for detection of cortisol in saliva could influence the present results.

## 5. Conclusions

The results of this study show no correlation between OLP and stress. The lack of a correlation in this study could be due to the small number of the patients and potential differences in cortisol level due to different times of awakening which could not be altered. According to the results of these and previously published data, further research is needed on a larger number of patients to determine the correlation between stress and the appearance of oral lichen planus.

## Figures and Tables

**Table 1:** Salivary cortisol levels in patients with oral lichen planus (OLP) and healthy subjects.

	Erosive/Atrophic OLP	Reticular OLP	Healthy Subjects	*p* *
Salivary cortisol level (µg/dL) (median (range))	0.108 (0.064–0.336)	0.141 (0.007–0.486)	0.106 (0.013–0.292)	>0.05

* Kruskal–Wallis test.
